# Genome Sequence of a Chromium-Reducing Strain, *Bacillus cereus* S612

**DOI:** 10.1128/genomeA.01392-15

**Published:** 2015-12-10

**Authors:** Dongping Wang, Hakim Boukhalfa, Doug S. Ware, Paul W. Reimus, Hajnalka E. Daligault, Cheryl D. Gleasner, Shannon L. Johnson, Po-E Li

**Affiliations:** a Earth Systems Observations (EES-14), Earth and Environmental Sciences Division, Los Alamos National Laboratory, Los Alamos, New Mexico, USA; b Bioenergy and Biome Sciences, Biology Sciences Division, Los Alamos National Laboratory, Los Alamos, New Mexico, USA

## Abstract

We report here the genome sequence of an effective chromium-reducing bacterium, *Bacillus cereus* strain S612. The size of the draft genome sequence is approximately 5.4 Mb, with a G+C content of 35%, and it is predicted to contain 5,450 protein-coding genes.

## GENOME ANNOUNCEMENT

Potassium dichromate utilization as an additive in the water used for cooling at many Department of Energy (DOE) power plants has resulted in significant releases of chromium contamination into the environment. Liquid effluents released at TA-03 to the Sandia Canyon in Los Alamos, NM, from 1956 to 1972 have resulted in the release of approximately 31,000 to 72,000 kg of hexavalent chromium (1). The chromate anion is relatively mobile, toxic, and carcinogenic; therefore, it is of major environmental concern. Fortunately, the reduction of hexavalent chromium to the trivalent form reduces both the toxicity and mobility of chromium in the environment (2–4). The reduction of hexavalent chromium can be achieved anaerobically (5) and aerobically (6) by indigenous bacteria. A range of bacterial species capable of reducing hexavalent chromium have been isolated and characterized. *Bacillus cereus* comprises a metabolically versatile, spore-forming, and environmentally ubiquitous bacterial species. *B. cereus* strains isolated from wastewater, sediments, and soils demonstrate promising chromium-reducing traits (7–9).

In this work, we report the sequencing of a *B. cereus* strain that reduces chromate under aerobic conditions. The bacterium was isolated from groundwater obtained from a monitoring well located at the center of the chromium plume located in Sandia Canyon in the Los Alamos area. The strain grows on an LB plate containing 10 g/liter potassium dichromate. It also exhibits a promising ability for Cr(VI) reduction. Here, we announce the draft genome sequence of *B. cereus* S612. Analysis of the genome will provide information about the genetic bases that establish its high-level chromium tolerance and reduction.

Genome sequencing was performed using a MiSeq sequencer with 250-bp read chemistry (Illumina), as described previously (10–12). Briefly, genomic DNA from *B. cereus* S612 was isolated from an overnight culture using the UltraClean microbial DNA isolation kit (Mo Bio, USA). The genomic library was constructed using 10 ng of genomic DNA fragmented by the Covaris E210 instrument and prepared using the NEBNext Ultra DNA library preparation kit for Illumina (NEB). The sequencing run was set up using a version 3 MiSeq sequencing reagent kit to generate 2 × 250-bp reads.

The filtered sequences were *de novo* assembled using IDBA and Velvet and computationally shredded into 1.5-kbp overlapping shreds (13, 14). All shreds were integrated using Phrap (15). Twenty-eight contigs with an average of 260× genome coverage were obtained. The contigs ranged from 504 to 1,000,381 bp in size. The assembled data were annotated using an Ergatis-based (16) workflow, with minor manual curation. The genome size was found to be 5,403,786 bp, comprising 5,450 protein-coding genes and 120 RNA-coding genes. The strain S612 genome harbors genes encoding multidrug efflux pumps and reductases (7) that are potentially related to chromium resistance and reduction. *B. cereus* S612 is currently explored as a biological control agent for hexavalent chromium removal in the groundwater system.

### Nucleotide sequence accession number.

This whole-genome shotgun project of *B. cereus* strain S612 has been deposited at DDBJ/EMBL/GenBank under the accession no. LJXA00000000. The version described in this paper is the first version.
